# Electron Crystallography Reveals Plasticity within the Drug Binding Site of the Small Multidrug Transporter EmrE

**DOI:** 10.1016/j.jmb.2008.01.056

**Published:** 2008-04-04

**Authors:** Vladimir M. Korkhov, Christopher G. Tate

**Affiliations:** MRC Laboratory of Molecular Biology, Hills Road, Cambridge, CB2 0QH, UK

**Keywords:** multidrug resistance, membrane protein, structure, conformation

## Abstract

EmrE is a Small Multidrug Resistance transporter (SMR) family member that mediates counter transport of protons and hydrophobic cationic drugs such as tetraphenylphosphonium (TPP^+^), ethidium, propidium and dequalinium. It is thought that the selectivity of the drug binding site in EmrE is defined by two negatively charged glutamate residues within a hydrophobic pocket formed from six of the α-helices, three from each monomer of the asymmetric EmrE homodimer. It is not apparent how such a binding pocket accommodates drugs of various sizes and shapes or whether the conformational changes that occur upon drug binding are identical for drugs of diverse chemical nature. Here, using electron cryomicroscopy of EmrE two-dimensional crystals we have determined projection structures of EmrE bound to three structurally different planar drugs, ethidium, propidium and dequalinium. Using image analysis and rigorous comparisons between these density maps and the density maps of the ligand-free and TPP^+^-bound forms of EmrE, we identify regions within the transporter that adapt differentially depending on the type of ligand bound. We show that all three planar drugs bind at the same pocket within the protein as TPP^+^. Furthermore, our analysis indicates that, while retaining the overall fold of the protein, binding of the planar drugs is accompanied by small rearrangements of the transmembrane domains that are different to those that occur when TPP^+^ binds. The regions in the EmrE dimer that are remodelled surround the drug binding site and include transmembrane domains from both monomers.

## Introduction

EmrE is an *Escherichia coli* Small Multidrug Resistance (SMR) protein that couples inward translocation of two protons with the counter-transport of a broad range of hydrophobic cations, such as tetraphenylphosphonium (TPP^+^), ethidium and methyl viologen.^1,2^ EmrE contains 110 amino acids that form four transmembrane (TM) α-helices.^3,4^ A low resolution (7.5 Å) projection structure^3^ and 3D structure of EmrE in complex with its substrate TPP^+^ have been determined by electron cryomicroscopy (cryo-EM) of two-dimensional (2-D) crystals.^5^ The structure revealed that EmrE is composed of eight helices arranged as an asymmetric dimer within the membrane. The dimeric arrangement of EmrE was corroborated by analytical ultracentrifugation and substrate binding studies.^6,7^ EmrE has been proposed to adopt a dual topology configuration in the bacterial membrane, based on the low-resolution structural information,^5^ GFP/PhoA fusion-based topology mapping and mutagenesis,^8^ although this has been disputed based mainly on cross linking data.^9^ The low-resolution cryo-EM structure revealed the position of TPP^+^ in the middle of an eight-helix transmembrane bundle.^5^ Based upon cryo-EM data and evolutionary constraints, a model for EmrE has been built;^10^ the model is supported by extensive mutagenesis studies that identified residues likely to be involved in substrate binding.^1,11,12^

To achieve multi-specific drug binding and transport EmrE has evolved a hydrophobic pocket with a pair of negatively charged glutamic acid residues (Glu14), which are crucial for direct transporter–ligand interactions, substrate translocation and coupling to the transmembrane proton gradient.^13^ By analogy with the soluble multidrug binding proteins QacR^14^ and BmrR,^15^ the binding site of EmrE could be assumed not to require a perfect stereochemical fit with the drugs, which is believed to be a common principle in all multidrug binding proteins.^13^ Binding of hydrophobic cations to EmrE is therefore thought to be via non-specific hydrophobic interactions and the ionic forces between the two E14 residues and the delocalized positive charge of the drug. Such a ‘simple’ binding pocket would reduce the evolutionary constraints on the protein and ensure broad substrate specificity. However, it is not clear how the binding of EmrE can accommodate drugs of different sizes and shapes, and whether different conformational changes are required.

We have, therefore, used cryo-EM of 2-D crystals to determine the projection structures of EmrE bound to three different substrates to address this question. Comparison of these projections with those obtained previously, with and without TPP^+^ bound, shows that the multidrug binding site of EmrE is flexible and adopts distinct conformations dependent on the nature of the substrate.

## Results

### Crystallization of EmrE with various substrates

**Fig. 1:**
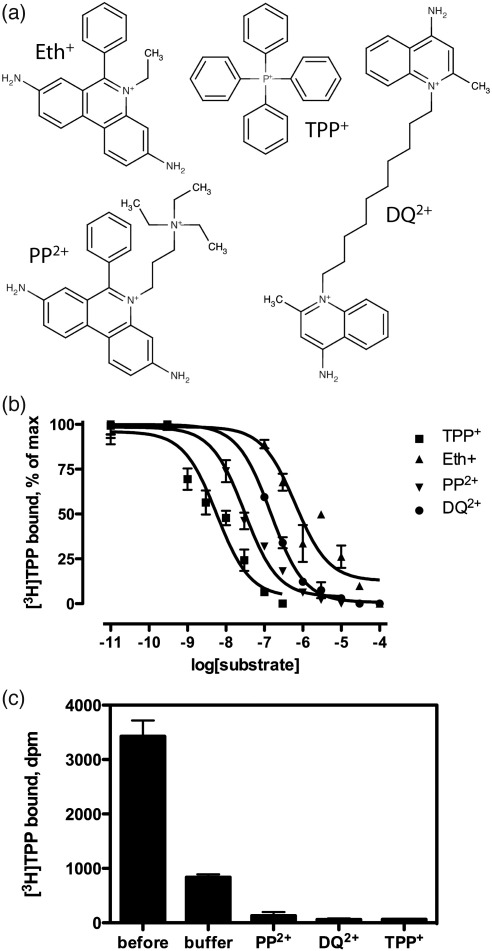
Substrates used for EmrE 2-D crystallization. (a) Chemical structures of the three substrates: ethidium (Eth^+^), propidium (PP^2+^) and dequalinium (DQ^2+^); tetraphenylphoshonium (TPP^+^) is shown for comparison. (b) Competition curves confirm that the chosen substrates have micromolar or submicromolar affinities for EmrE. The data shown are from a single representative [^3^H]TPP^+^ displacement experiment, performed on detergent-solubilized EmrE containing a final [^3^H]TPP^+^ concentration of 6 nM. IC_50_ values were used to estimate the K_i_ of the competitors using Cheng–Prusoff equation,^23^ with a K_d_ of TPP^+^ binding of 2.4 nM: IC_50_ values were 1.8 μM, 33 nM and 147 nM for Eth^+^, PP^2+^ and DQ^2+^ respectively, corresponding to K_i_ values of 1.0 ± 0.9 μM, 19 ± 4 nM and 83 ± 9 nM. (c) Exchange of [^3^H]TPP^+^ bound to 2-D crystals for unlabeled substrates. Residual [^3^H]TPP^+^ bound to EmrE was measured (*n* = 3) after a 1000-fold dilution and 5 h incubation with and without propidium and dequalinium; error bars represent the SEM.

EmrE was previously crystallized by dialysis in the presence of lipids to form 2-D crystals either with or without bound TPP^+^, a high-affinity substrate^3,5,16^ that binds to EmrE with an affinity of 2 nM^7^. All the previously obtained projection maps and the low-resolution 3D structure of EmrE were determined using these 2-D crystals. To gain insights into the mechanisms underlying the substrate promiscuity of EmrE, it was critical to gather structural data on its complexes with other drugs. Therefore, three different substrates were chosen to investigate properties of the EmrE drug binding pocket. Ethidium (Eth^+^), propidium (PP^2+^) and dequalinium (DQ^2+^) all differ structurally from TPP^+^ by being predominantly planar molecules rather than tetrahedral (Fig. 1a).^23^ Eth^+^ contains a planar triple aromatic ring system and, although PP^2+^ is very similar to Eth^+^, it contains a second tertiary amine and therefore carries a 2+ charge. Finally, DQ^2+^ has two planar cationic ‘head groups’ separated by an aliphatic C10 linker.

Radioligand competition binding assays showed that each of the ligands possesses micro- or submicromolar affinity for EmrE, with K_i_ values for Eth^+^, PP^2+^ and DQ^2+^ of 1.0 μM, 19 nM and 83 nM, respectively (Fig. 1b). Crystallisation trials were therefore performed in the presence of either excess Eth^+^, PP^2+^ or DQ^2+^ to obtain 2-D crystals of the various EmrE–substrate complexes. Crystals of the Eth^+^-bound form of EmrE were of reasonable quality, although they were not as good as TPP^+^–EmrE crystals. However, the inclusion of either PP^2+^ or DQ^2+^ during 2-D crystallizations resulted in either extremely poorly ordered crystals or large unordered vesicles, both unsuitable for structure determination. We speculated that the reason for this disorder was due to the amphipathic nature of PP^2+^ and DQ^2+^, which resulted in them partitioning into the lipid bilayer and disrupting the molecular interactions necessary for 2-D crystal formation. We therefore had to use substrate exchange to substitute the TPP^+^ in 2-D crystals of the TPP^+^–EmrE complex for either PP^2+^ or DQ^2+^. Ligand exchange was accomplished by dialyzing 2-D crystals of TPP^+^-bound EmrE against a 1000-fold excess of buffer containing either PP^2+^ or DQ^2+^ for 5 h. The extent of the ligand exchange was estimated by using [^3^H]TPP bound to EmrE 2-D crystals during the exchange reaction. Only 1–3% of radioactive TPP^+^ remained bound to the 2-D crystals of EmrE after a 5 h incubation following a 1000-fold dilution of the 2-D crystals when the dialysis buffer contained either PP^2+^ or DQ^2+^ (Fig. 1c), suggesting that the structural data would be dominated (> 97%) by contributions from EmrE bound to the exchanged ligand. Under the conditions used for the ligand exchange, equilibrium was nearly reached, but longer incubations could have resulted in a further deterioration of crystallinity, so exchange reactions were limited to 5 h.

### Projection structures of ethidium-, propidium- and dequalinium-bound EmrE

**Fig. 2:**
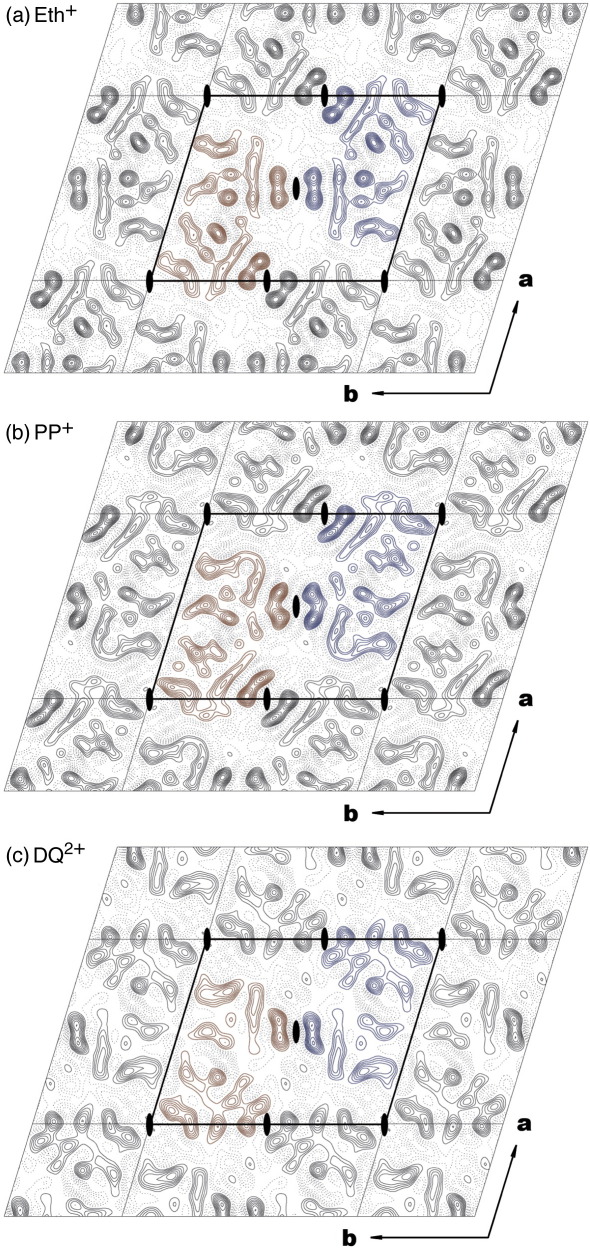
The projection maps of planar drug-bound EmrE. (a–c) The 2-D crystals of EmrE bound to an indicated drug were subjected to cryo-EM and image analysis, as described under “Materials and Methods” (Eth^+^,ethidium; PP^2+^, propidium; DQ^2+^, dequalinium). The projection maps were plotted using NPO, with contours ranging from − 500 to 500, with a step of 25; the 0 contour is omitted. The crystallographic asymmetric unit consists of a tetramer composed of two asymmetric dimers related by an in-plane 2-fold axis. One tetramer is coloured red and the other blue.

The crystals obtained in the presence of Eth^+^ were smaller than the TPP^+^-bound crystal form, but reasonably well ordered, judged by optical diffraction of the cryo-EM micrographs and further image processing. The projection structure was obtained by merging data from the 12 best images. The resolution of the projection structure extended to 7 Å, comparable to that of the previously determined EmrE projections with and without TPP^+^ (Fig. 2a, Table 1).

The crystals that underwent ligand exchange (PP^2+^- and DQ^2+^-bound crystal forms) were morphologically indistinguishable from the parental TPP^+^-bound EmrE 2-D crystals. However, image processing revealed that the substrate exchange procedure led to distortions within the crystals and, subsequently, to loss of their quality. Most of the imaged EmrE 2-D crystals suffered fragmentation and were rendered unsuitable for structural work. We hypothesise that the disruption of the crystal lattice was largely due to partitioning of the PP^2+^ or DQ^2+^ into the lipid bilayer and disrupting EmrE crystal contacts, although we cannot fully discount contributions from an additional conformational change accompanying the binding of PP^2+^ or DQ^2+^. Only a small proportion of crystalline areas within a few of the observed 2-D crystals remained sufficiently ordered for further processing. To obtain the projection structures for PP^2+^- and DQ^2+^-bound forms of EmrE, 7 and 5 images were merged, respectively (Fig. 2b and c, Table 1). Despite the strong effects of ligand exchange on the crystallinity of the specimen, the PP^2+^–EmrE and DQ^2+^–EmrE projection maps contained reliable information at 8–9 Å (Table 1): the effective resolution thresholds for both projection maps were 8.2 Å. All three projection structures obtained from Eth^+^-, PP^2+^- and DQ^2+^-bound EmrE crystals had *p*2 symmetry, identical to the TPP^+^-bound crystals.

### Averaged projection structures of EmrE bound to mono- and bivalent planar substrates

**Fig. 3:**
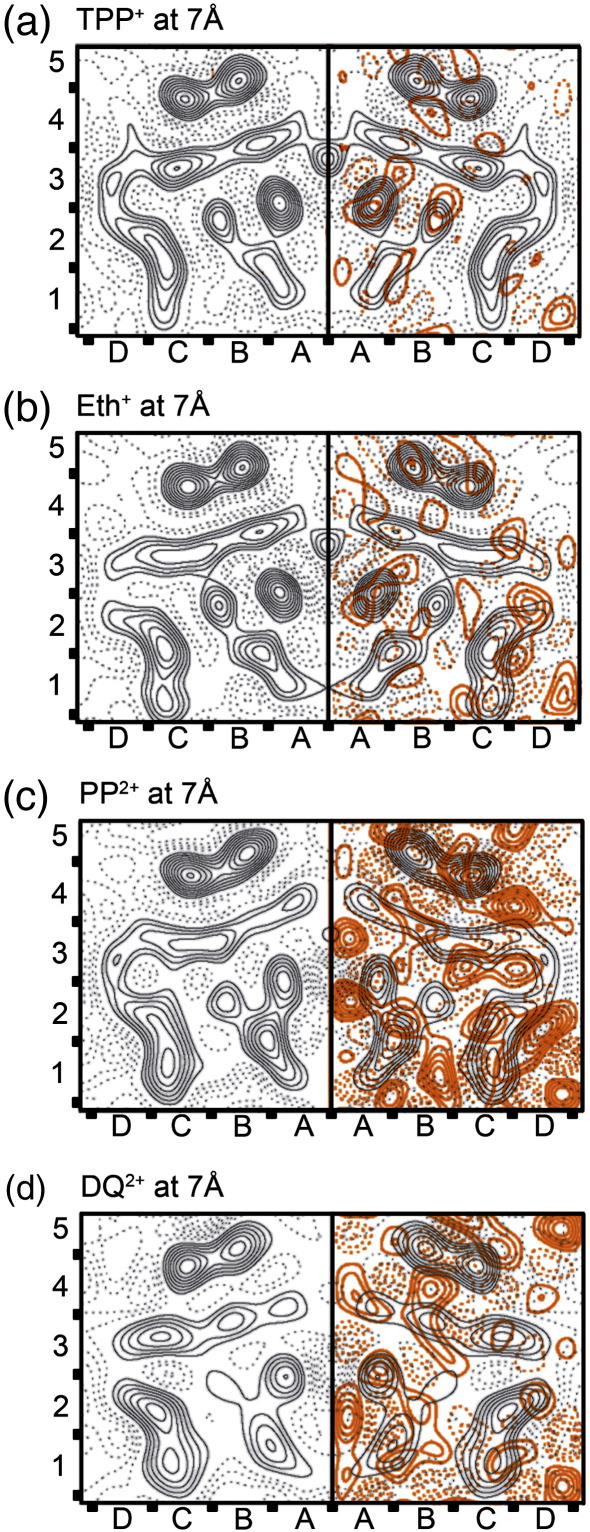
Averaged projection maps of EmrE with bound TPP^+^ and three different planar drugs. (a–d) Maps of the density corresponding to the EmrE tetramer bound to indicated drugs, after image interpolation and averaging of the two halves of the non-crystallographic tetramer. The red coloured contours in the right half of each map show the differences between the two dimers within a tetramer (the difference maps were obtained by subtraction of the mirror images, as described in “Materials and Methods”). The coordinate grid imposed on the projection maps (*x*-axis: a–d; *y*-axis: 1–5) is arbitrary, although the x = 0 line coincides with the in-plane non-crystallographic 2-fold axis used in the averaging procedure.

At the resolution of the projection maps obtained, all secondary structure features seen in the higher resolution projections of EmrE were clearly resolved (Fig. 2). The repetitive unit in the crystals was the crystallographic tetramer (coloured either blue or red in each of the maps in Fig. 2), but previous work shows that the minimal functional unit for substrate binding is the dimer.^7^ Careful inspection clearly revealed small differences between the two halves of the tetramer related by two fold non-crystallographic symmetry of EmrE in each of the projection maps. These discrepancies between the dimers were more pronounced in the density maps obtained with PP^2+^ and DQ^2+^, where only a limited amount of data was available. To improve further the resolution of the asymmetric dimer, the two dimers in the crystallographic tetramer were averaged using the MRC software for electron micrograph analysis,^17^ as described in “Materials and Methods”. The resulting averaged maps calculated from the data and merged to 7 Å are shown in Fig. 3. Evident from the red contours in Fig. 3, the asymmetry within the crystallographic tetramer was highest in the PP^2+^-bound EmrE projection map, although the data extended to about the same resolution as that for the DQ^2+^-bound form (Table 2, “RMS mirr.”).

**Fig. 4:**
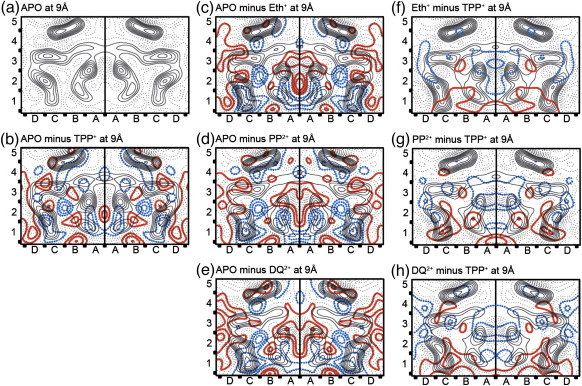
Comparison between the averaged projection structures of ligand-free and planar drug-bound EmrE. (a) Density map of the apo form of EmrE, truncated to 9 Å resolution, calculated from the apo-EmrE projection obtained by imaging 2-D crystals of space group c222.^3^ (b) Projection map of TPP^+^–EmrE, truncated to 9 Å, is contoured in black. Solid red contours correspond to the densities above 0 level in the difference map made by subtracting the TPP^+^–EmrE density from the apo-EmrE density; the 0 contour is omitted. Dotted blue contours correspond to negative densities in the same difference map. (c–e) Projection maps of Eth^+^-, PP^2+^- and DQ^2+^-bound EmrE, respectively, and corresponding apo-EmrE difference maps are contoured as in b. Resolution of each map 9 Å. (f–h) Comparison between the TPP^+^- and the planar drug-bound EmrE. Projection maps at 9 Å of Eth^+^-, PP^2+^- and DQ^2+^-bound EmrE, respectively, are contoured in black. Solid red and dotted blue contours correspond to positive and negative densities in the difference maps between planar ligand-bound EmrE and TPP^+^–EmrE maps (as in c–e).

The three projection structures of EmrE were superficially similar to each other and to those obtained previously (Fig. 3). In order to quantitatively assess the similarity between all ligand-bound (TPP^+^ and planar drugs) and ligand-free (apo-) EmrE projection maps, we also calculated the averaged dimer TPP^+^–EmrE map from the *p*2-symmetrised projection structure^16^ (as described under “Materials and Methods”; Fig. 4b, Table 2). The apo form (Fig. 4a) did not require averaging, because the projection maps from which it was derived were in space group *c*222 that contains a crystallographic in-plane 2-fold relating the two asymmetric dimers in the tetramer.^16^ Thus, interpolation and averaging served two purposes, improving the signal/noise level in the resulting projection structures and, more importantly, allowing the direct comparisons of the projection maps calculated from 2-D crystals with either *p*2 (TPP^+^, Eth^+^, PP^2+^ and DQ^2+^) or *c*222 symmetry (apo-EmrE).

The resolution at which the comparisons of all maps could be performed was limited by the resolution of the DQ^2+^-bound EmrE map (effective resolution threshold of 8.2 Å). To choose an optimal resolution, we merged and averaged all five maps at 7, 8, 9 and 10 Å. We then determined the “RMS difference” (the RMS deviation of the total density from the average density in the map; Table 2) for difference maps between each projection map and the TPP^+^–EmrE. We found that the RMS deviations, and therefore the experimental error, were lowest at 9 Å for all planar ligand-bound EmrE projection maps. Therefore, all maps were compared and cross-correlated at 9 Å resolution. The maps of TPP^+^–EmrE, Eth^+^–EmrE and apo-EmrE contained structural information below that threshold, and were compared separately at 7 Å resolution.

### Detailed comparisons between projection maps

**Fig. 5:**
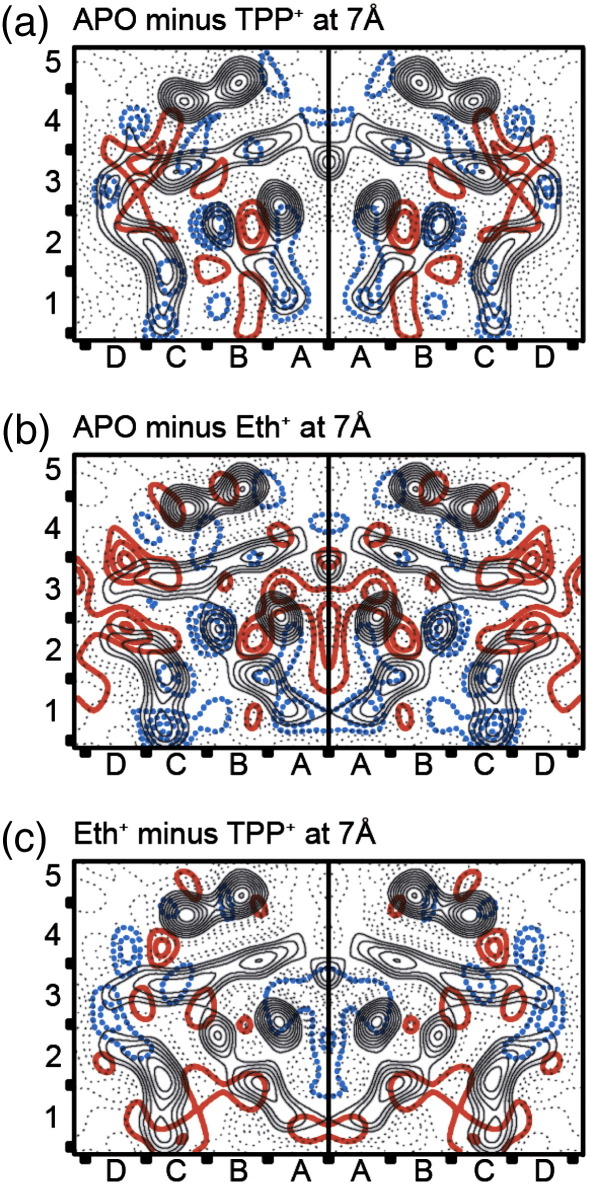
Comparison of the three different conformational states of EmrE at higher (7 Å) resolution. (a, b) The projection maps of EmrE bound to either TPP^+^ or Eth^+^ and the differences between these maps and the apo-EmrE are shown with the same representation as in Fig. 4b and c. (c) Differences between Eth^+^ and TPP^+^-bound EmrE are shown with the same representation as in Fig. 4f. Resolution of all the maps is 7 Å.

Comparisons were made between all the projection maps and plotted at either 9 Å resolution (Fig. 4) or, where the data allowed, at 7 Å resolution (Fig. 5). Table 3 provides the cross-correlation coefficients for all the comparisons. From these data it is clear that there are three distinct projection structures, namely apo-EmrE, TPP^+^-bound EmrE and planar drug-bound EmrE. The differences between apo-EmrE and TPP^+^–EmrE have been previously described^16^ and are reproduced in Fig. 4b, with the large negative density peak at the B2/C2 boundary representing bound TPP^+^. Figure 4c–e show similar negative peaks, which are likely to represent either Eth^+^, PP^2+^ or DQ^2+^ in each case. In addition, there are density differences in each of the different density maps for Eth^+^, PP^2+^ and DQ^2+^ in the C1 area, suggestive of a movement of this helix, as seen upon TPP^+^ binding, although there appears to be an increase in the magnitude of the movement when planar drugs are bound, compared to TPP^+^ (Fig. 4f–h).

Additional changes are seen in the areas A3, D3 and D4 of the 7 Å resolution map ‘Eth^+^ minus TPP^+^’ (Fig. 5c). These changes are associated with the helices that form the substrate binding pocket, whereas there appear to be fewer and smaller density changes associated with the pair of helices in squares B&C, 4&5. These two helices may represent helix 4, one from each monomer, and are notable in that they are about 9 Å apart centre to centre along their whole length and so probably represent a strong and stable association; in addition, being separated from the ligand binding pocket, it is unlikely they are contributing to any conformational change during ligand binding.

## Discussion

Versatile recognition of diverse chemical structures is a feature of the multidrug binding proteins and transporters. Most of what we know about the structural basis of multi-specific drug recognition comes from the structural studies on the soluble multidrug binding proteins, such as BmrR and QacR,^14,15^ and from the X-ray structures of the multidrug transporter of the RND family member, AcrB^20^ (several other putative multidrug transporter structures have been solved, albeit without any substrates bound^18,19^). The structures of these proteins revealed large drug binding sites containing discrete subsites in which different drugs can bind.^14,15,20^ In some cases, binding of several drugs was shown to occur simultaneously, leading to a ‘cross-talk’ between the ligands and their binding sites.^20,21^ Importantly, in all cases where multidrug binding proteins or transporters were co-crystallized with the drugs, recognition of different drugs by the same binding site was associated with little, if any, difference in the conformational state of the protein.^13^

The drug binding site of EmrE is formed by the transmembrane domains of the protein. This puts several evolutionary constraints on the EmrE drug binding site: (i) there must be at least two orientations of the substrate binding site (inward- and outward-facing orientation), with different affinities for the substrates, allowing substrate translocation across the bacterial inner membrane; (ii) inherently, this implies a certain degree of flexibility of the protein; (iii) specificity for multiple drugs must be combined with strong coupling to the H^+^-gradient, preventing ‘leaky’ passage of ions or small molecules. Maintaining these three conditions would present a challenge for a small protein like EmrE with a large pocket containing multiple discrete binding sites for different classes of drugs.

Here, using cryo-EM we show that, to accommodate drugs with planar and tetrahedral geometry, EmrE undergoes distinct conformational rearrangements. The structural changes are similar for mono- and bivalent planar drugs (Eth^+^ and PP^2+^, respectively). We are able to identify the position of the bound ligand in all three new projection structures at 9 Å resolution (confirmed for Eth^+^–EmrE down to 7 Å resolution), confirming that there are no subsites for different substrates in the EmrE binding site. Furthermore, we identify three regions surrounding the drug binding site, which change in response to binding of planar drugs (Eth^+^, PP^2+^ and DQ^2+^) in a way different from the changes upon TPP^+^ binding. Remodelled regions of the protein include transmembrane helices in area A2 and B3, as well as the junction between helices at the boundary between areas 2 and 3. The peak corresponding to movement of the helix in area C1 is broadened in the planar drug–EmrE projection structures, although its position is not greatly changed. This may indicate intrinsic flexibility of this region of the protein, a property that may be necessary for drug binding and translocation across the membrane. The precise nature of the conformational changes will remain unresolved until 3D structures at higher resolution become available.

Our findings indicate that in contrast to other multidrug binding proteins, rather than having a large cavity with discrete niches for different substrates, EmrE has evolved a binding site that is remodelled dependent on the geometry of the substrate. Remodelling is achieved by subtle changes in the structure of the protein, resulting in altered positions of the transmembrane domains within the projection structures.

## Materials and Methods

### Protein expression and purification

Expression and purification of EmrE was performed as described previously.^3^ Briefly, *E. coli* membranes containing 6xHis-tagged EmrE were solubilized with dodecylmaltoside (DDM, Glycon, Germany). The protein was then purified using a 3-step procedure that included NiNTA-agarose (Qiagen), gel filtration (Superdex 200, Amersham) and anion exchange (PI, Poros). Purity of the protein preparations was assessed by SDS-PAGE.

### Radioligand binding assays

To quantify the efficiency of ligand exchange at the 2-D crystals, radioligand binding assays were used. For that purpose, the 2-D crystals obtained with TPP^+^ were subjected to dialysis against a low pH buffer (pH 5), to remove bound TPP^+^. A saturating amount of the resulting 2-D crystals was mixed with [^3^H]TPP^+^ (final concentration 400 nM; Amersham) in the crystallization buffer (Tris pH 7.5, NaCl 100 mM, ethylenediaminetetraacetic acid (EDTA) 1 mM). The mix was diluted 1000-fold with the crystallization buffer in the absence and in the presence of propidium iodide (15 μM) or dequalinium (50 μM). After 5 h incubation at 22 °C, 1 ml of the diluted mix was filtered though GF/B filter (Millipore) and washed with ice-cold buffer. Liquid scintillant was added and radioactivity bound to the filter was counted. Non-specific binding was determined by adding 100 μM cold TPP^+^ (Sigma). Competition binding assays were performed as described previously.^6^

### 2-D crystallization

Crystallization of tubular crystals of EmrE in complex with ethidium bromide was performed essentially as that for the TPP^+^-bound form.^3^ EmrE at concentration of 0.5–1 mg/ml was mixed with dimyristoylphosphatidylcholine (DMPC; Avanti Polar Lipids) at lipid to protein ratios of 0.3–0.4. Crystallization was performed in a dialysis cassette (Slide-a-lyzer, 10 kDa cut-off, 0.1–0.5 ml, Pierce) for a week, with a daily buffer exchange (pH 7.5) with a buffer containing 1 mM ethidium bromide.

### Ligand exchange

To obtain the crystals of EmrE bound to propidium iodide and dequalinium, the TPP^+^-bound EmrE crystals were employed. A suspension of the TPP^+^–EmrE crystals (100 μl, 1 mg/ml protein concentration, 200 nM TPP^+^) was dialysed for 5 h at 22 °C against a 1000-fold excess of the same buffer containing the desired ligand (50 μM propidium or 15 μM dequalinium; Sigma). The concentrations of propidium and dequalinium were well above the determined K_i_ values (2632 times and 181 times, respectively) for binding to EmrE. A 1000-fold dilution of the TPP^+^ in the crystal suspension dilutes it to 10-fold below its K_d_ to optimise ligand exchange. In Fig. 1c, this would mean equilibrium would have been reached if the amount of ^3^H-TPP^+^ left bound was 3.5 dpm, whereas after exchange the amount left bound when exchanged for propidium was 105 dpm and 35 dpm in the presence of unlabelled TPP^+^ or dequalinium. Longer incubations were avoided to prevent deterioration of the crystals.

### Electron microscopy

Crystals were deposited onto glow-discharged carbon-coated electron microscopy grids, blotted, washed with a solution containing 2% glucose, blotted again, and plunged into liquid nitrogen. The frozen grids were loaded onto a liquid nitrogen-cooled Gatan 626 cryostage and analysed by low-dose cryo-EM techniques (10–15 e Å^− 2^) on a Technai F20 electron microscope at an accelerating voltage of 200 keV. Images were collected with flood beam illumination at a magnification of 50,000×–58,000×. The quality of the images was assessed by optical diffraction. The best images were digitized using the MRC KZA scanner with a 6 μm step size.^22^ Image processing was performed using the MRC package. Projection structures were built according to previously described procedures, using MRC software.^17^ Averaging of the asymmetric dimers within the crystallographic tetramer was done using INTERPO, a program for image interpolation. In brief, the *p*2 maps were rotated by approximately 17° (slightly different angles were used for each map; Table 2), to enable assignment of an orthogonal (90°) coordinate system to each projection map. This allowed the direct comparison of maps from either *p*2 or *c*222 crystal forms. An area corresponding to the EmrE crystallographic tetramer was cut out from each map using LABEL, and then the same program was used to create a mirror image of the cut out image. TWOFILE was then used to calculate difference maps between the two mirror images. Iterative adjustment of the angle of rotation and the position of the cut out region was performed until the lowest RMS deviation of the mirror difference map was reached (“RMS mirr.”; Table 2). The procedure was repeated with all projection maps merged at 7, 8, 9 and 10 Å resolution. TWOFILE was then used to compare the averaged projection maps at corresponding resolutions; each projection map was scaled to obtain minimal RMS of the difference map (“RMS diff.”; Table 2). Cross-correlation of the difference maps was performed with the mean density values, using the equation: σ = (A·B)/*sqrt*(A^2^·B^2^), where A and B are mean densities of the two correlated difference maps; multiplication of images required for the cross-correlation calculation was done using TWOFILE.

## Figures and Tables

**Table 1:** Electron crystallographic data

Crystal form	EmrE-ethidium		EmrE-propidium		EmrE-dequalinium	
Plane group symmetry	*p*2		*p*2		*p*2	
Unit cell dimensions						
a (Å)	74.57 ± 0.57		69.33 ± 0.51		70.05 ± 0.45	
b (Å)	89.93 ± 1.48		83.38 ± 0.46		83.92 ± 1.26	
γ (deg.)	106.69 ± 1.16		106.05 ± 0.54		105.75 ± 0.45	
No. images	12		7		5	
Range of defocus (Å)	4113_10488		6202_16069		5614_15612	
No. unique reflections	191		126		102	
Total observations	1694		805		615	
Overall phase residual	27.70		40.99		27.79	
Temperature factor B_xy_ (Å)	347		288		344	

Resolution range (Å)	No. of unique reflections	Phase residuals	No. of unique reflections	Phase residuals	No. of unique reflections	Phase residuals

∞–15.81	40	12.99	32	25.30	31	11.06
15.81–11.18	39	21.36	25	37.72	26	22.36
11.18–9.13	38	22.15	29	24.77	26	24.10
9.13–7.90	38	30.66	16	63.29	11	68.20
7.90–7.07	34	56.40	22	73.17	7	72.83
7.07–6.45	33	74.63	11	64.41	4	52.72
6.45–5.98	26	89.67	16	71.99	7	96.03

**Table 2:**
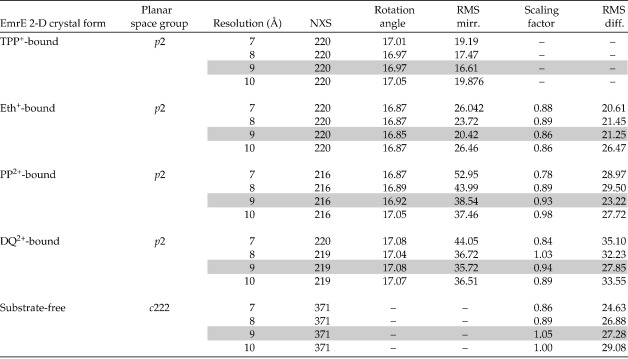
Statistics for map averaging and comparisons

For definitions of the abbreviations and the calculation of the numbers, see Materials and Methods.

**Table 3:**
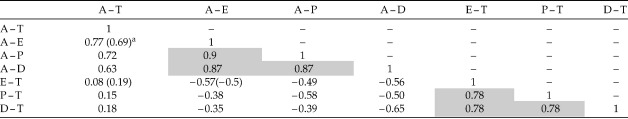
Cross-correlation coefficients for the comparison of all possible combinations of difference maps

Abbreviations used here: A, T, E, P and D stand for apo-EmrE, TPP^+^, ethidium, propidium and dequalinium, respectively; thus ‘A-T’ indicates the difference density map calculated by subtracting the density for TPP^+^-bound EmrE from the density for apo-EmrE. ^a^Cross-correlation coefficients determined using the difference maps calculated at 7 Å resolution are shown in parentheses.
